# Urine NGAL as an early biomarker for diabetic kidney disease: accumulated evidence from observational studies

**DOI:** 10.1080/0886022X.2019.1617736

**Published:** 2019-06-04

**Authors:** Xing-Yao Tang, Jian-Bo Zhou, Fu-Qiang Luo, Yi-Peng Han, Wei Zhao, Zong-Li Diao, Mei Li, Lu Qi, Jin-Kui Yang

**Affiliations:** a Beijing Tongren Hospital, Capital Medical University, Beijing, China;; b Department of Endocrinology, Beijing Tongren Hospital, Capital Medical University, Beijing, China;; c Department of Epidemiology, School of Public Health and Tropical Medicine, Tulane University, New Orleans, LA, USA;; d Department of Geriatrics, Beijing Tongren Hospital, Capital Medical University, Beijing, China;; e Division of Nephrology, Beijing Friendship Hospital, Capital Medical University, Beijing, China;; f Division of Education, Beijing Tongren Hospital, Capital Medical University, Beijing, China;; g Beijing Key Laboratory of Diabetes Research and Care, Beijing, China

**Keywords:** NGAL, diabetic kidney disease, diagnosis

## Abstract

**Objectives:** Urine neutrophil gelatinase-associated lipocalin (NGAL) was found to increase in diabetic kidney disease (DKD). However, the clinical value of urine NGAL as diagnostic indicators in DKD remains to be clarified.

**Methods:** Relevant studies were systematically retrieved from PubMed, Embase, Web of Science, and the Cochrane Library. Stratified analyses and regression analyses were performed.

**Results:** Fourteen studies with 1561 individuals were included in our analysis, including 1204 cross-sectional participants and 357 cohort participants. For the cross-sectional studies, the pooled sensitivity and specificity of NGAL in the diagnosis of DKD were 0.82 (95% confidence interval (CI): 0.75–0.87) and 0.81 (95% CI: 0.68–0.90), respectively. The pooled diagnostic odds ratio was 19 (95% CI: 11–33), and the overall area under the curve was 0.88 (95% CI: 0.84–0.90). For the cohort studies, the pooled sensitivity and specificity of NGAL in the diagnosis of DKD were 0.96 (95% CI: 0.91–0.98) and 0.89 (95% CI: 0.84–0.92), respectively. The overall area under the curve was 0.98, indicating good discriminative ability of NGAL as biomarkers for DKD.

**Conclusions:** Urine NGAL, as the early diagnostic marker of DKD, might have the high diagnostic value, especially in cohort studies.

## Introduction

Diabetic kidney disease (DKD) is considered one of the primary microvascular complications of diabetes and arguably the most devastating one, given that those with kidney disease predominantly account for the increased morbidity and mortality among diabetic patients [1]. Essentially, an early detection is of pivotal importance in improving clinical management. Now microalbuminuria and the decrease in GFR level are wildly accepted as standards for diagnosing DKD, albeit accumulated evidence has shown that their predictive value is limited. From recent studies, however, the concept of ‘diabetic tubulopathy’ has emerged and the name Diabetic Nephropathy is gradually replaced by DKD [2]. Diabetic tubulopathy refers to impaired reabsorption of filtered proteins which may play a role as an initiator, driver or contributor in the early pathogenesis of DKD [3].

Lately, one biomarker which has been regarded as the direct indicator of proximal tubule injury during early DKD occurs: neutrophil gelatinase-associated lipocalin (NGAL) [4,5]. It is a small, 25-kDa protein that belongs to the lipocalin protein family released from neutrophil and many epithelial cell types including kidney tubular cells [6]. It represents the tubular mass function and produces rapidly and massively during the response to tubular injury [7].

However, with massive studies, the diagnostic power of NGAL as a biomarker for DKD remains unknown. To address this, we accumulated evidence of observational studies to assess the diagnostic accuracy of NGAL.

## Materials and methods

### Study inclusion

The PubMed and EMBASE databases and Web of Science and Cochran Library were searched for relevant studies published until March 2019. The search terms of the PubMed used both text words and MeSH subject headings and included (‘Diabetic nephropathy’ or ‘Nephropathies, Diabetic’ or ‘Nephropathy, Diabetic’ or ‘Diabetic Nephropathy’ or ‘Diabetic Kidney Disease’ or ‘Diabetic Kidney Diseases’ or ‘Kidney Disease, Diabetic’ or ‘Kidney Diseases, Diabetic’ or ‘Diabetic Glomerulosclerosis’ or ‘Kimmelstiel-Wilson Syndrome’ or ‘Kimmelstiel Wilson Syndrome’ or ‘Syndrome, Kimmelstiel-Wilson’ or ‘Kimmelstiel-Wilson Disease’ or ‘Kimmelstiel Wilson Disease’ or ‘Nodular Glomerulosclerosis’ or ‘Glomerulosclerosis, Nodular’ or ‘Glomerulosclerosis, Diabetic’ or ‘Intracapillary Glomerulosclerosis’) AND (‘Neutrophil-gelatinase-associated lipocalin’ or ‘NGAL’). For the Embase, Web of Science and Cochran Library, we used (‘Diabetic Kidney Diseases’ or ‘Diabetic nephropathy’) AND (‘Neutrophil-gelatinase-associated lipocalin’ or ‘NGAL’).

The search strategy was supplemented by inspecting the references of the including articles. This report was conducted according to the recommendations of the preferred reporting items for systematic reviews and meta-analyses (PRISMA) guidelines [8].

### Inclusion and exclusion criteria

Studies were considered for inclusion if they (1) were original articles recently published in Chinese or English, (2) provided sufficient data to construct a 2 × 2 table for calculating the diagnostic accuracy, (3) measured the urine NGAL levels, (4) enrolled healthy controls or patients with urinary albumin but not diagnosed of DKD, and (5) used the diagnosis of diabetes established by WHO, renal damages distinguished by albumin:creatinine ratio (ACR) or 24 h urinary albumin quantification. Exclusion criteria were as follows: (1) the publication was a review, case report, or letter to the editor; (2) there was no control group; (3) the authors could not provide valid data after being contacted; (4) it was not specified for the staging of diabetic nephropathy; (5) the non-DKD group included only the healthy controls.

### Data extraction and quality assessment

**Figure 1.**
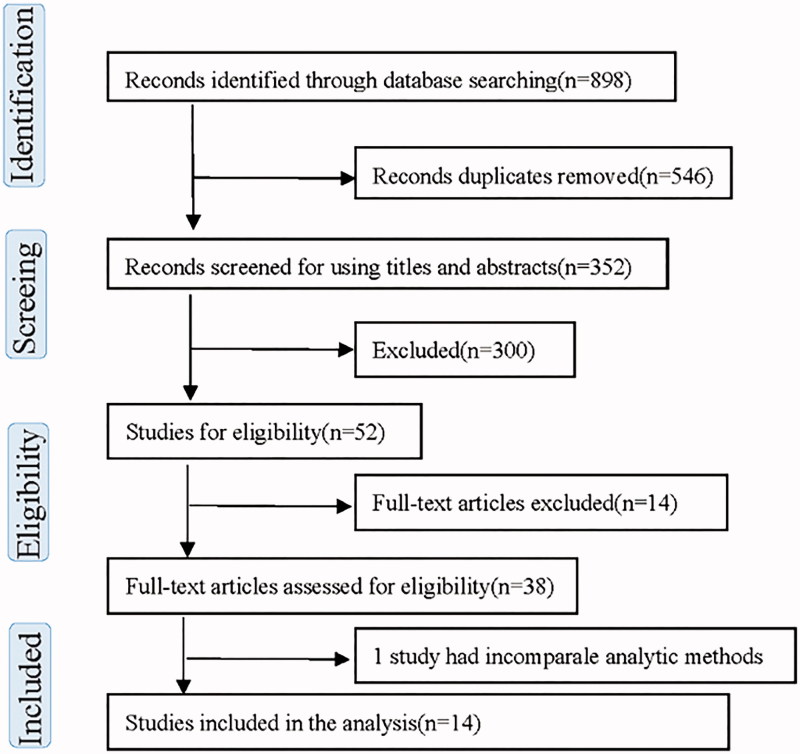
Chart of the diagram in this study.

Two investigators (X.Y.T. and F.Q.L.) independently extracted the data from the 14 enrolled studies, using a standard form that included study, year, country of origin, testing method, number of cases, control type, and cutoff value. Two investigators (X.Y.T. and Y.P.H.) independently utilized the quality assessment of diagnostic accuracy studies-2 (QUADAS-2) [9] to assess the risk of bias for the enrolled studies. If there was disagreement, the investigators discussed the study with the other authors to arrive at a consensus (Figure 1).

### Statistical analysis

The statistical analyses were performed with Meta-Disc version 1.4 (Universidad Completeness, Madrid, Spain). Pooled sensitivity, specificity, positive likelihood ratio (LR) and negative LR were calculated to assess the efficacy of NGAL in sifting out DKD patients from all diabetic patients. The diagnostic odds ratio (DOR) and AUC of the summary receiver operator characteristic (SROC) curve were used to evaluate the overall performance of the diagnostic test. The heterogeneity of the included studies caused by the threshold effect was quantified by Spearman’s correlation analysis. Also, the non-threshold effect was assessed by using a chi-squared test and *I*^2^ statistics. A chi-square test of *p* < .10 or *I*^2^ > 50% indicated the existence of heterogeneity caused by a non-threshold effect. In addition, meta-regression was used to find the possible sources of heterogeneity caused by the non-threshold effect. Fagan’s nomogram was employed to calculate the post-test probabilities. Potential publication bias was evaluated by Deeks’ funnel plot asymmetry test, and in this test *p* < .05 was considered statistically significant.

## Results

### Study selection and study characteristics

The search strategy identified 898 potentially relevant records, of which 546 were excluded as they were duplicates. The remaining 352 manuscripts were sent to title and abstract screening. We then removed 300 publications because they were reviews, letters, conference abstracts, or unrelated studies. Therefore, 52 articles were eligible for full-text review and data assessment. Thirty-eight articles were finally excluded due to unavailable data for constructing a 2 × 2 contingency table, and the remaining 14 studies were enrolled in the meta-analysis [4,10–18]. In the 1561 research individuals adopted, 11 of them were cross-sectional studies [4,10,12,13,15,16,18] with 1204 individuals and three of them were cohort studies [11,14,17] with 357 individuals. A flowchart demonstrating the study selection process is illustrated in Table 1.

**Table 1.** Characteristics of included studies.

Study	Year	Country	Test method	Study design	TP	FP	FN	TN	Cutoff (ng/ml)	Non-DKD						DKD					
										Total (M/F)	Average age	SBP (mmHg)	DBP (mmHg)	HbA1c (%)	Duration (years)	Total (M/F)	Average age	SBP (mmHg)	DBP (mmHg)	HbA1c (%)	Duration (years)
Abd El Dayem et al. [33]	2017		ELISA	Prospective cohort	20	19	0	9	94000000000	28						20					
Assal et al. [10]	2013	Egypt	ELISA	Cross-sectional	33	2	17	18	8.8	20 (10/10)	51.3 ± 6.3	131.0±5.7	83.6±4.5	7.6±0.7	5.7±2.2	25 (14/11)	52.9 ± 6.8	141.7±6.6	87.2±6.7	8.0±1.3	8.7±3.7
Bolignano et al. [4]	2009	Italy	ELISA	Cross-sectional	30	0	10	34	22	16 (7/9)	49 ± 8	128 ± 36	82 ± 31	6.9±1.1	12 (9–15)	40 (19/21)	51 ± 10	141 ± 35	87 ± 34	7.3±1.2	15 (13–18)
Chen et al.	2018	China	Immunoturbidimetry	Cross-sectional	95	30	25	150	15.4	180 (95/85)	27–69	NA	NA	NA	NA	120 (64/56)	26–68	NA	NA	NA	NA
Chen et al.	2016	China	ELISA	Cross-sectional	40	6	16	105	177.6	56	61.5 ± 9.3	135.2 ± 15.9	74.9 ± 12.6	NA	NA	167	NA	NA	NA	NA	NA
Hafez et al. [12]	2015	Egypt	Immunonephelometric method	Cross-sectional	10	13	2	25	11.75	12	13.84 ± 4.00	110.00 ± 13	70.79 ± 11.18	8.29 ± 1.29	8.57 ± 0.53	38	13.84 ± 4.00	121.25 ± 18.11	79.58 ± 13.39	8.29 ± 1.29	8.57 ± 0.53
Huang et al.	2017	China	Immunoturbidimetry	Cross-sectional	88	13	13	34	25	47	NA	NA	NA	NA	NA	101	NA	NA	NA	NA	NA
Hosny et al. [13]	2018	Egypt	ELISA	Cross-sectional	38	0	2	20	0.038	20	58.18 ± 13.98	NA	NA	9.17 ± 1.09	7.92 ± 4.95	20	58.18 ± 13.98	NA	NA	9.20 ± 1.81	7.92 ± 4.95
Kaul et al. [14]	2018	India	ELISA	Prospective cohort	103	0	5	36	21.31	36 (22/14)	NA	NA	NA	NA	NA	108 (67/41)	NA	NA	NA	NA	NA
Sueud et al.	2019	Australia	ELISA	Cross-sectional	71	11	4	4	21.4	30 (16/14)	45.3 ± 6.9	NA	NA	NA	5.3 ± 7.2	60 (21/39)	NA	NA	NA	NA	NA
Vijay et al. [16]	2018	India	ELISA	Cross-sectional	52	18	11	45	146.28	63 (35/28)	49.2 ± 11.37	NA	NA	7.42 ± 0.68	3.95 ± 2.54	63 (33/30)	54.25 ± 13.06	NA	NA	9.18 ± 1.72	9.11 ± 3.94
Yıldırım et al. [17]	2015	İstanbul	ELISA	Prospective cohort	10	4	1	61	36.3	65	NA	NA	NA	NA	NA	11	NA	NA	NA	NA	NA
Zeng et al. [18]	2017	China	ELISA	Cross-sectional	28	13	14	91	85	104 (60/44)	57.6 ± 12.7	116.5 ± 10.7	77.3 ± 4.1	8.5 ± 1.8	9.3 ± 2.3	42 (24/18)	55.7 ± 15.7	119.5 ± 8.4	77.6 ± 5.7	7.8 ± 2.3	14.0 ± 3.6
Zylka et al.	2018	Poland	Chemiluminescent microparticle immunoassay	Cross-sectional	15	24	4	37	14.3	61 (32/29)	59±11	NA	NA	6.30 (5.90–7.80)	NA	19 (6/13)	67±12	NA	NA	7.35 (6.30–8.40)	NA

TP: true positive; FP: false positive; FN: false negative; TN: true negative; NA: not applicable.

Table 1 summarizes the details and main characteristics of the 11 cross-sectional studies, which were published between 2009 and 2019. The sample size varied from 50 to 300. Of the 11 studies, five studies enrolled patients from Asia, three were performed Africa, and two was done in Europe, and one study was on Oceanian patients.

Table 1 also summarizes the details and main characteristics of the three cohort studies, which were published between 2009 and 2018. The sample size varied from 48 to 198. The three studies were from Asia, Africa, and Europe.

### Quality assessment

Quality assessment results of the studies are shown in Supplement 1 using the QUADAS-2 evaluation tool. The quality of the included studies varied from moderate to high.

### Diagnostic accuracy analysis

**Figure 2.**
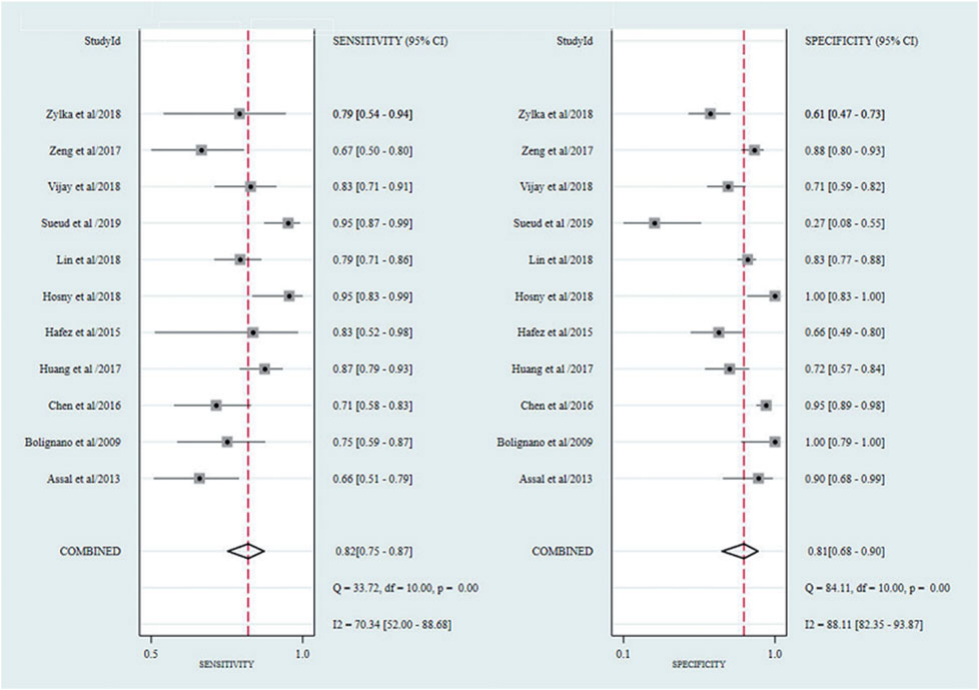
The forest plot of sensitivity and specificity of cross-sectional studies.

**Figure 3.**
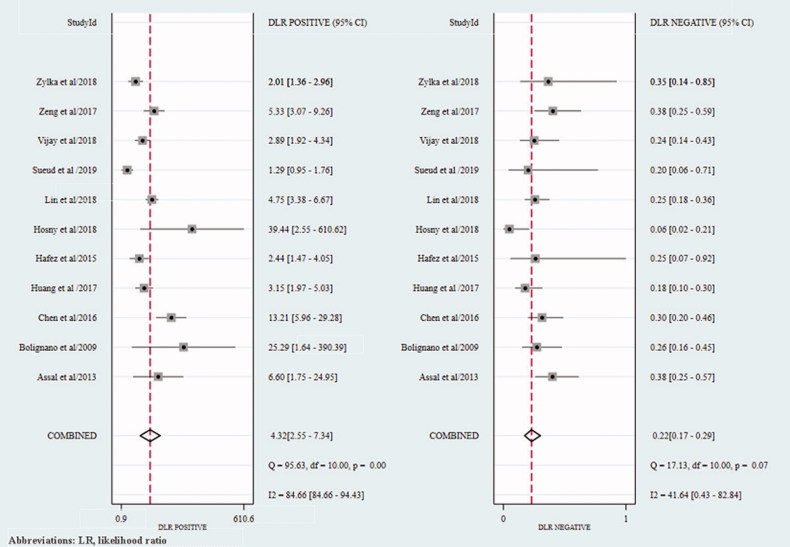
The forest plot of positive LR and negative LR of cross-sectional studies. LR: likelihood ratio.

**Figure 4.**
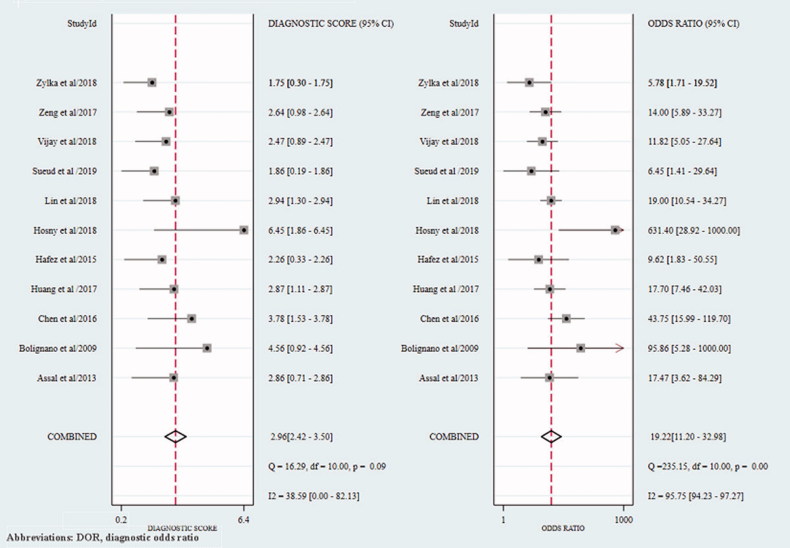
The forest plot of DOR of cross-sectional studies. DOR: diagnostic odds ratio.

**Figure 5.**
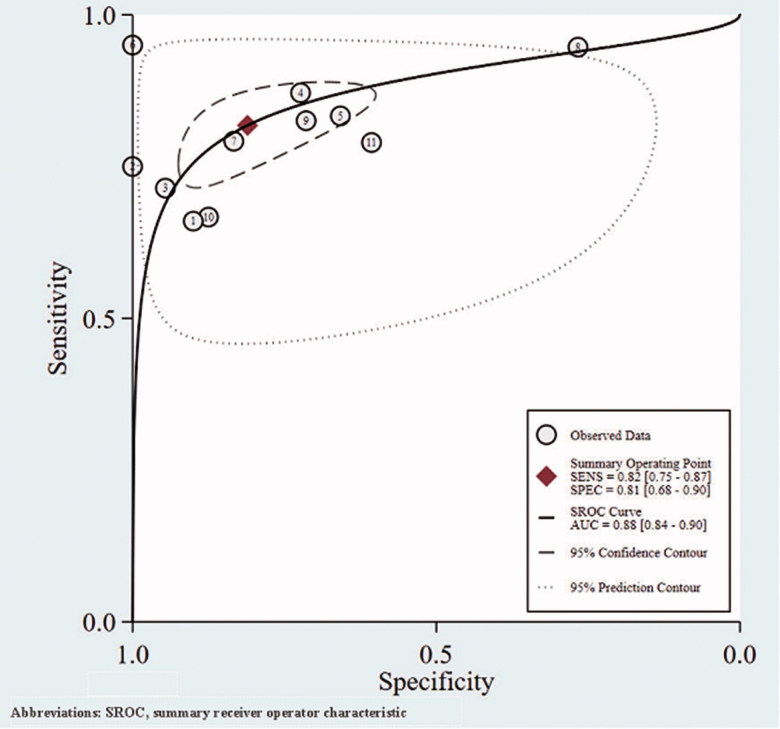
The SROC curve of cross-sectional studies. SROC: summary receiver operator characteristic.

In the cross-sectional studies, the pooled sensitivity for the studies included in the final meta-analysis was 0.82 (95% confidence interval (CI): 0.75–0.87), and the pooled specificity was 0.81 (95% CI: 0.68–0.90) (Figure 2). In addition, the overall positive LR and negative LR were 4.3 (95% CI: 2.5–7.3) and 0.22 (95% CI: 0.17–0.29), respectively (Figure 3). The overall diagnostic accuracy was assessed by pooled DOR (19, 95% CI: 11–33) and by AUC of the SROC curve (0.88, 95% CI: 0.84–0.90) (Figures 4 and 5). Significant heterogeneity across the studies was detected according to the *I*^2^ value of DOR (95.75%).

As for the cohort studies, the pooled sensitivity and the pooled specificity for the studies included in the final meta-analysis were 0.96 (95% CI: 0.91–0.98) and 0.89 (95% CI: 0.84–0.92) (Supplement 3A and B). In addition, the overall positive LR was 14.00 (95% CI: 0.21–953.35) and the overall negative LR 0.06 (95% CI: 0.03–0.12), respectively (Supplement 3C and D).

To find the origin of the heterogeneity, we performed a series of analyses, including threshold effect, subgroup analysis, and meta-regression.

### Diagnostic threshold effect

Threshold effect is a pivotal source of heterogeneity in diagnostic tests. It is caused by the differences in sensitivity and specificity. One good way to assess the threshold effect is by using Spearman’s correlation coefficient of sensitivity and specificity. Our analysis showed that the Spearman correlation coefficient was 0.39 (*p* = .24) and 0.50 (*p* = .67) respectively for the 11 cross-sectional studies and the three cohort studies, indicating the absence of a threshold effect.

### Subgroup analyses and meta-regression analysis

Through the regression analysis of the cross-sectional studies, we observed there were two variables which significantly affected results of the sensitivity (*p* < .05): (1) description of methods of patients selection (yes or no) and (2) method of NGAL measurements (ELISA or immunoturbidimetry).

In studies with description of methods of patient selection, the pooled sensitivity was 0.80 (95% CI: 0.73–0.88), the pooled specificity was 0.78 (95% CI: 0.64–0.92); and in studies without description of methods of patient selection, the pooled sensitivity was 0.85 (95% CI: 0.77–0.94), the pooled specificity was 0.85 (95% CI: 0.71–0.99). In studies by ELISA to measure NGAL, the pooled sensitivity was 0.80 (95% CI: 0.73–0.88), and its pooled specificity was 0.86 (95% CI: 0.75–0.96); in studies by immunoturbidimetry to measure NGAL, the pooled sensitivity was 0.84 (95% CI: 0.75–0.93), and its pooled specificity was 0.72 (95% CI: 0.51–0.92) (Supplement 2).

### Publication bias

**Figure 6.**
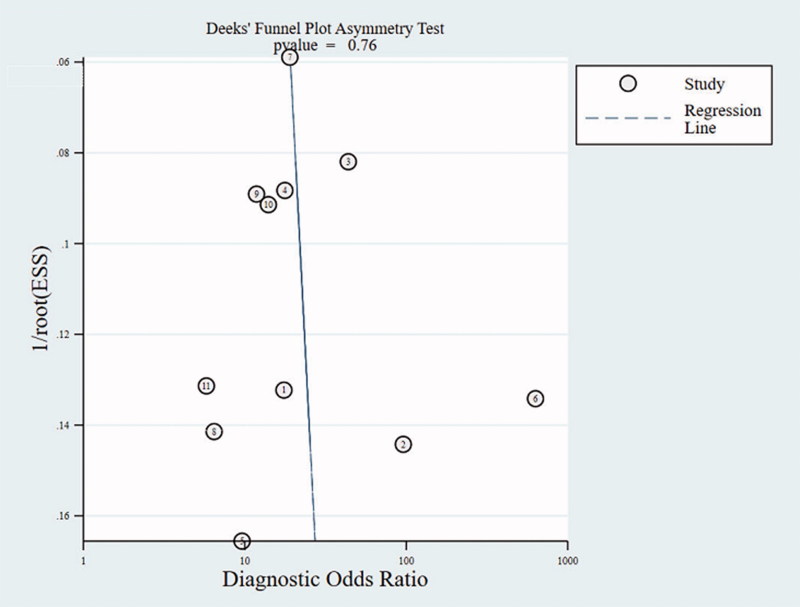
Deed’s funnel plot of cross-sectional study.

The publication bias of the included cross-sectional studies was checked by Deeks’ funnel plot asymmetry test, and the result is presented in Figure 6. A statistically non-significant value (*p* = .76) in the funnel plot indicated no potential publication bias.

## Discussion

In this study, accumulated evidence has shown the diagnostic value of urine NGAL in DKD, which indicated that urine NGAL could distinguish patients with DKD from the controls. The results of cross-sectional studies showed that the pooled sensitivity and specificity were 82% and 81%, respectively, which also means a rate of missed diagnosis (18%) and misdiagnosis (19%). Moreover, the cohort studies provided a pooled sensitivity of 96% and a pooled specificity of 89%, and also with a rate of missed diagnosis (4%) and misdiagnosis (11%).

Human NGAL protein is a 25-kDa protein covalently bound to neutrophil gelatinase, and it was first found in secondary granules of human neutrophils. In addition, NGAL is an iron-trafficking protein which is secreted from the ureteric bud in the embryonic kidney and regulate the primordial mesenchymal cell – the renal tubular epithelial progenitor. This is achieved by forming the NGAL:siderophore:Fe3+ complex, and this iron carrier is essential for cell differentiation and nephron formation [19]. Moreover, the release of NGAL possesses kidney-protective activities as well. Its induction is one of the noticeable preservation of kidney function, reduced apoptosis, and an enhanced proliferative response [20]. Previous studies elucidated that NGAL mRNA is robustly expressed in several matured human tissues, including neutrophils, liver, kidney, etc. [21]. Under the circumstance of kidney injury, the rapid and also massive upregulated synthesis of NGAL protein in the distal tubule [22] and proximal tubule in ischemic renal damage [23], yield the quick increase of urinary NGAL level [24]. Studies on patients with diabetes have also shown that an increased NGAL expression can be detected in those with a normal or slight increase in albuminuria, suggesting a possible tubulopathy in the early stage of DKD [4,5,13].

The LR can fully reflect the diagnostic value of the screening test, which is a stable indicator of the diagnostic test and is not affected by the prevalence rate. The greater the positive likelihood ratio (PLR) of the diagnostic test is, the greater possibility the test positive person will actually become ill. When PLR > 10, the diagnostic test has high efficacy. The combined PLR value of the cross-sectional studies and the cohort studies were 4.3 and 14.0, respectively. Both two results were of moderately diagnostic value. The DOR combines the strengths of sensitivity and specificity, and expresses the diagnostic performance as an independent indicator. A higher DOR value represents a better discriminatory test performance [25]. The pooled DOR of cross-sectional studies was 19 (95% CI: 11–33), which showed a high diagnostic significance of NGAL for patients with diabetes. In addition, the AUC of SROC was used to assess the overall test performance. An AUC with a value ranging between 0.93 and 0.96 suggests an excellent diagnostic value of NGAL, and a value from 0.75 to 0.92 means an acceptable diagnostic value of it [26,27]. Our results showed that urine NGAL had acceptable diagnostic accuracy in diabetic patients with an AUC of 0.88 (95% CI: 0.84–0.90) of the cross-sectional studies. In the cohort studies, the AUC was 0.98, which indicated the higher diagnostic value.

Threshold effect is a pivotal source of heterogeneity in diagnostic tests. It is caused by the differences in sensitivity and specificity. One good method to assess the threshold effect is by using Spearman’s correlation coefficient of sensitivity and specificity. Our analysis showed that the Spearman correlation coefficient in total among the 11 cross-sectional studies and three cohort studies was 0.39 (*p* = .24) and 0.50 (*p* = .67), indicating the absence of a threshold effect. Additionally, two Spearman correlation coefficients suggested that the threshold effect was not the cause of heterogeneity in the meta-analysis. Furthermore, we used a stratified analysis to examine the heterogeneity in prespecified subgroups. Meta-regression analysis indicated that the heterogeneity was related to two sides: one was whether a study had description of the methods in patient selection (*p* = .001), the other was method of NGAL measurements (ELISA or immunoturbidimetry) (*p* = .01). Subgroup analysis showed that studies with description of methods of patient selection had the pooled sensitivity of 80% and the pooled specificity of 78%. As for the studies by ELISA to measure NGAL, the pooled sensitivity and specificity were 80% and 86%, respectively. Their diagnostic values were comparable to that of all included studies.

In spite of our efforts to accomplish a comprehensive and accurate analysis, this meta-analysis still has certain limitations. First, all of the enrolled subjects were in English or Chinese, which decreased the applicability of the results across different literatures. Second, the selected diagnostic threshold value of NGAL was quite different, while there was no threshold effect in the included literature, which did not affect the overall evaluation result. Third, this accumulated analysis was not registered, which maybe result in lack of transparency. Additionally, subgroup analysis and publication bias were not performed because few cohort studies were included. Sample sizes in cohort studies were relatively small, as a result, small-study effects might present [28].

In the past, a large number of relevant studies have been conducted. They were not included in this meta-analysis because we failed to extract data from them. In Wu et al. [29] studies of 293 type 2 DKD’s patients and non-diabetic controls, their results indicated that urine levels of NGAL and RBP may independently associate with albuminuria in T2DKD and may serve as novel biomarkers for the identification of T2DKD. Four other studies [32] have reached similar conclusions, which are consistent with the results of this study.

## Conclusions

In summary, accumulated evidence from observational studies demonstrated the efficacy of urine NGAL as the early diagnostic marker of DKD, especially in cohort studies. However, the diagnostic value of urine NGAL in DKD still needs to be further explored.

## Supplementary Material

Supplemental File
