# Supplementary material for: Urine NGAL as an early biomarker for diabetic kidney disease: accumulated evidence from observational studies
Source: Ren Fail. 2019 Jun 4;41(1):446–54. doi: 10.1080/0886022X.2019.1617736 (PMC6566833; doi:10.1080/0886022X.2019.1617736)
Supplement: Supplemental File [file IRNF_A_1617736_SM3477.docx]

Search strategy：

The search was undertaken on March, 12 2019.

The search was conducted through in the PubMed, Embase, Web of science and Cochran Library.

Search terms were as follows:

1. PubMed:

‘Diabetic nephropathy’ or ‘Nephropathies, Diabetic’ or ‘Nephropathy, Diabetic’ or ‘Diabetic Nephropathy’ or ‘Diabetic Kidney Disease’ or ‘Diabetic Kidney Diseases’ or ‘Kidney Disease, Diabetic’ or ‘Kidney Diseases, Diabetic’ or ‘Acute Kidney Injury, Diabetic’ or ‘Acute Kidney Injuries, Diabetic’ or ‘Diabetic Acute Kidney Injury’ or ‘Diabetic Acute Kidney Injuries’ or ‘Diabetic Glomerulosclerosis’ or ‘Kimmelstiel-Wilson Syndrome’ or ‘Kimmelstiel Wilson Syndrome’ or ‘Syndrome, Kimmelstiel-Wilson’ or ‘Kimmelstiel-Wilson Disease’ or ‘Kimmelstiel Wilson Disease’ or ‘Nodular Glomerulosclerosis’ or ‘Glomerulosclerosis, Nodular’ or ‘Glomerulosclerosis, Diabetic’ or ‘Intracapillary Glomerulosclerosis’

AND

‘Neutrophil-gelatinase-associated lipocalin’ or ‘NGAL’

2. Embase, Web of science and Cochran Library:

‘Diabetic Kidney Diseases’ or ‘Diabetic nephropathy’

AND

‘Neutrophil-gelatinase-associated lipocalin’ or ‘NGAL’

| **Supplement.1** Risk of Bias and Applicability Judgments in QUADAS-2 | | | | | | | | | | | | | | | | | | | | | | | |
| --- | --- | --- | --- | --- | --- | --- | --- | --- | --- | --- | --- | --- | --- | --- | --- | --- | --- | --- | --- | --- | --- | --- | --- |
|  | | | | | | | | | | | | | | | | | | | | | | | |
| Study | |  | | Vijay et al  2018 | Zeng et al 2017 | | Assal et al 2013 | Chen  et al 2018 | | Kaul et al 2017 | Bolignano et al 2009 | | Hafez et al 2015 | | Yıldırım et al 2015 | | Hosny et al 2018 | | Zylka et al 2018 | Sueud et al  2019 | Huang et al 2017 | Chen et al 2016 | Abd El Dayem  et al  2017 |
| Patient Selection | |  | |  |  | |  |  | |  |  | |  | |  | |  | |  |  |  |  |  |
|  | | 1_Signaling question1 | | Y | N | | N | N | | Y | N | | N | | Y | | N | | Y | Unclear | Y | Y | N |
|  | | 2_Signaling question2 | | N | N | | N | N | | N | N | | N | | N | | N | | N | N | N | N | N |
|  | | 3_Signaling question3 | | N | N | | N | N | | N | N | | N | | N | | N | | N | N | N | N | N |
|  | | 4_Risk of Bias | | UR | HR | | HR | HR | | HR | HR | | HR | | HR | | HR | | HR | HR | HR | HR | HR |
|  | | 5_Applicability | | HC | UC | | LC | LC | | UC | LC | | LC | | LC | | LC | | LC | LC | LC | LC | LC |
| Index Test | |  | |  |  | |  |  | |  |  | |  | |  | |  | |  |  |  |  |  |
|  | | 6_Signaling question1 | | N | N | | N | N | | N | N | | N | | N | | N | | N | N | N | N | N |
|  | | 7_Signaling question2 | | N | N | | N | N | | N | N | | N | | N | | N | | N | N | N | N | N |
|  | | 8_Risk of Bias | | HR | HR | | HR | HR | | LR | HR | | HR | | HR | | HR | | HR | HR | HR | HR | HR |
|  | | 9_Applicability | | HC | LC | | LC | LC | | HC | LC | | LC | | LC | | LC | | LC | LC | LC | LC | HR |
| Reference Standard | | | |  |  | |  |  | |  |  | |  | |  | |  | |  |  |  |  |  |
|  | | 10_Signaling question1 | | Y | Y | | Y | Y | | Y | Y | | Y | | Y | | Y | | Y | Y | Y | Y | Y |
|  | | 11_Signaling question2 | | Y | Y | | Y | Y | | Y | Y | | Y | | Y | | Y | | Y | Y | Y | Y | Y |
|  | | 12_Risk of Bias | | LR | LR | | LR | LR | | LR | LR | | LR | | LR | | LR | | LR | LR | LR | LR | LR |
|  | | 13_Applicability | | HC | HC | | HC | HC | | HC | HC | | HC | | HC | | HC | | HC | HC | HC | HC | HC |
| Flow and Timing | |  | |  |  | |  |  | |  |  | |  | |  | |  | |  |  |  |  |  |
|  | | 14_Signaling question1 | | Unclear | Y | | Y | Y | | Y | Y | | Y | | Y | | Y | | Y | Y | Y | Y | Y |
|  | | 15_Signaling question2~3 | | Y | Y | | Y | Y | | Y | Y | | Y | | Y | | Y | | Y | Y | Y | Y | Y |
|  | | 16_Signaling question4 | | N | Y | | Y | Y | | N | Y | | Y | | Y | | Y | | Y | Y | Y | Y | N |
|  | | 17_Risk of Bias | | HR | LR | | LR | LR | | HR | LR | | LR | | LR | | HR | | LR | LR | LR | LR | LR |
| LR:low risk | | UR:unclear risk | | HC:high concern | | |  | UC:unclear concern | | | | | | |  | |  | |  |  |  |  |  |
| 1_Was a consecutiveor random sample of patientsenrolled?2_Was a case – controldesignavoided?3_Did the study avoidinappropriate exclusions?4_Could the Selection of Patients Have Introduced Bias?5_Are There Concerns That the Included Patients and Setting Do Not Match the Review Question?6_Were the index test resultsinterpreted without knowledge of the results of the reference standard?7_If a threshold was used, was it prespecied?8_Could the Conduct or Interpretation of the Index Test Have Introduced Bias?9_Are There Concerns That the Index Test, Its Conduct, or Its Interpretation Differ From the Review Question?10_Is the referencestandard likely to correctlyclassifythe target condition?11_Werethe referencestandardresults interpretedwithout knowledgeof the resultsof the index test?12_Could the Reference Standard, Its Conduct, or Its Interpretation Have Introduced Bias?13_Are There Concerns That the Target Condition as Defined by the Reference Standard Does Not Match the Question?14_ Was therean appropriateinterval betweenthe index testand referencestandard?15_Did all patients receivethe same referencestandard?16_Were all patients included in the analysis?17_Could the Patient Flow Have Introduced Bias? | | | | | | | | | | | | | | | | | | | | | | | |
| **Supplement 2.** results of the sub-group analysis and the regression analysis. | | | | | | | | | | | | | | | | |  |  |  |  |  |  |  |
| Parameter | | category | | | LRTChi2 | | | Pvalue | | | I2 | | I2lo | | I2hi | |  |  |  |  |  |  |  |
| subject | | Yes | | | 4.89 | | | 0.09 | | | 59 | | 8 | | 100 | |  |  |  |  |  |  |  |
|  | | No | | | . | | | . | | | . | | . | | . | |  |  |  |  |  |  |  |
| index | | Yes | | | 0.89 | | | 0.64 | | | 0 | | 0 | | 100 | |  |  |  |  |  |  |  |
|  | | No | | | . | | | . | | | . | | . | | . | |  |  |  |  |  |  |  |
| reftest | | Yes | | | 2.11 | | | 0.35 | | | 5 | | 0 | | 100 | |  |  |  |  |  |  |  |
|  | | No | | | . | | | . | | | . | | . | | . | |  |  |  |  |  |  |  |
| participate | | Yes | | | 0.60 | | | 0.74 | | | 0 | | 0 | | 100 | |  |  |  |  |  |  |  |
|  | | No | | | . | | | . | | | . | | . | | . | |  |  |  |  |  |  |  |
| method | | Yes | | | 1.72 | | | 0.42 | | | 0 | | 0 | | 100 | |  |  |  |  |  |  |  |
|  | | No | | | . | | | . | | | . | | . | | . | |  |  |  |  |  |  |  |

Supplement 3. The forest plot of cohort studies


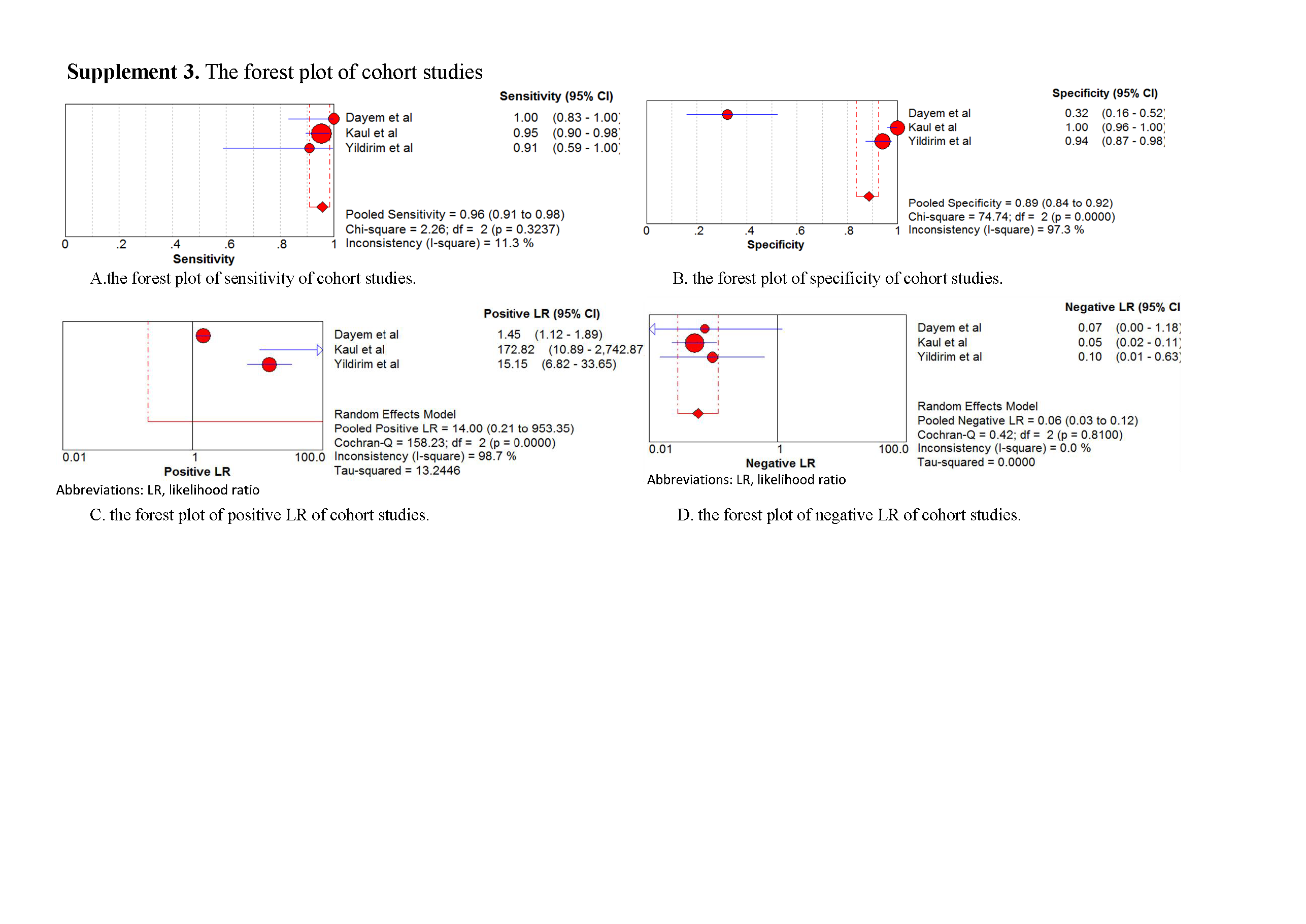


A. the forest of sensitivity of cohort studies


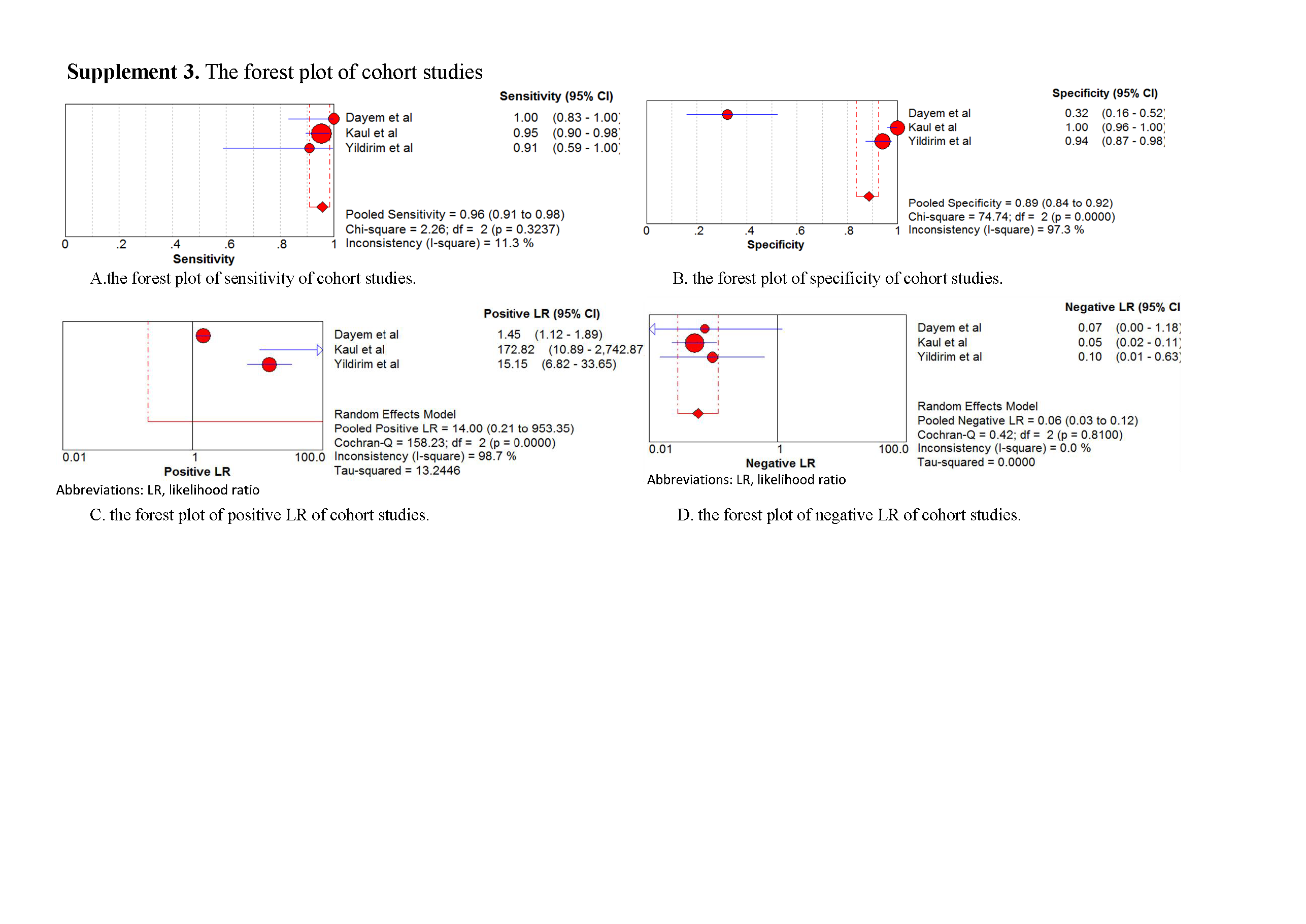


B. the forest plot of specificity of cohort studies


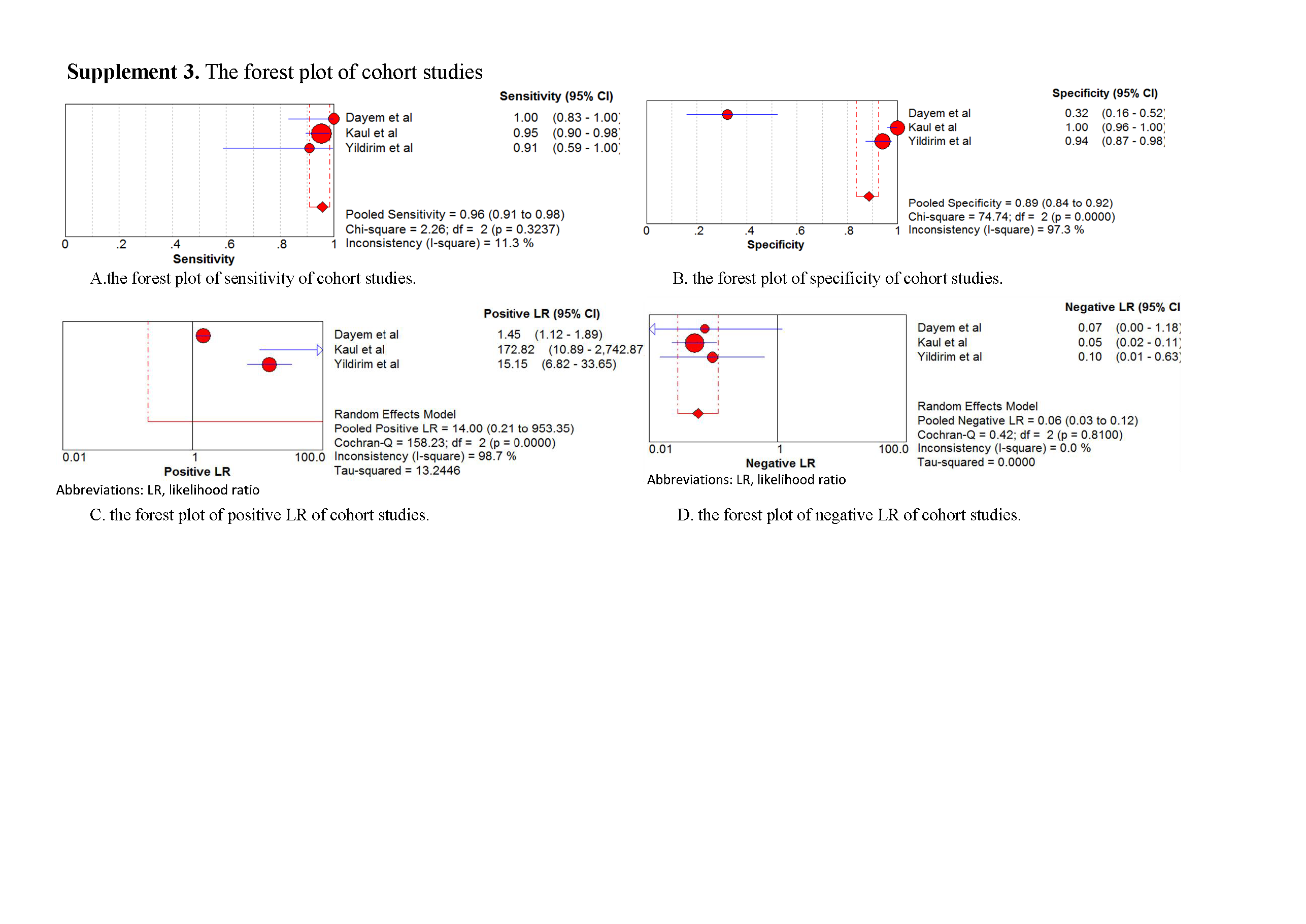


C. the forest plot of positive LR of cohort studies


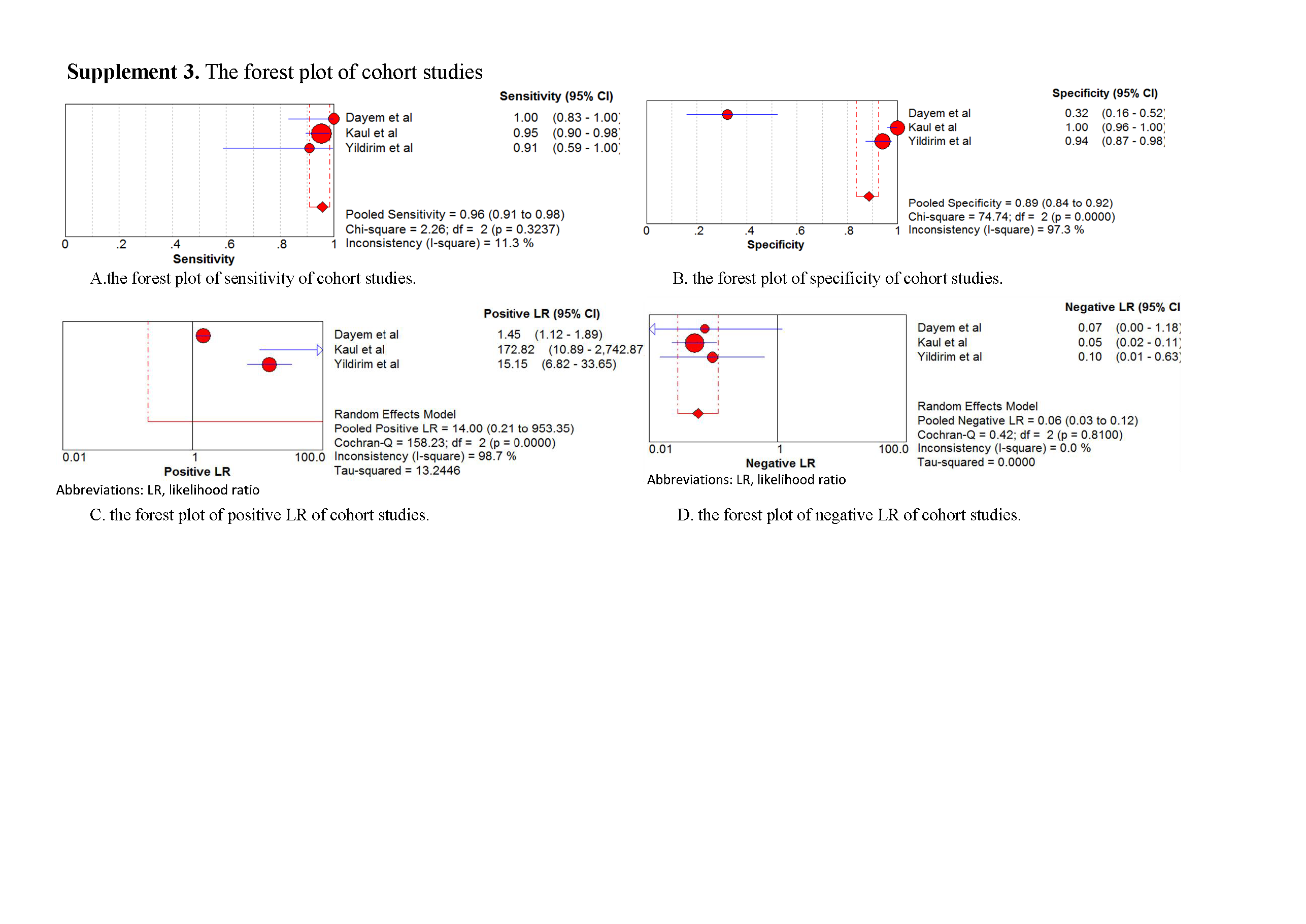


D. the forest plot negative LR of cohort studies

Avvreviations: LR, likelihood ratio
